# Changes in Number and Antibacterial Activity of Silver Nanoparticles on the Surface of Suture Materials during Cyclic Freezing

**DOI:** 10.3390/nano12071164

**Published:** 2022-03-31

**Authors:** Alexander Basov, Stepan Dzhimak, Mikhail Sokolov, Vadim Malyshko, Arkadii Moiseev, Elena Butina, Anna Elkina, Mikhail Baryshev

**Affiliations:** 1 Department of Fundamental and Clinical Biochemistry, Kuban State Medical University, 4 Mitrofan Sedina St., 350063 Krasnodar, Russia; son_sunytch79@mail.ru (A.B.); intro-2@rambler.ru (V.M.); 2 Department of Radiophysics and Nanothechnology, Kuban State University, 149 Stavropolskaya St., 350040 Krasnodar, Russia; jimack@mail.ru (S.D.); sokolovme@mail.ru (M.S.); baryshev_mg@mail.ru (M.B.); 3 Laboratory of Problems of Stable Isotope Spreading in Living Systems, Federal Research Center the Southern Scientific Center of the Russian Academy of Sciences, 41 Chekhov Ave., 344006 Rostov-on-Don, Russia; 4 Department of Organization and Support of Scientific Activities, Kuban State Agrarian University, 13 Kalinina St., 350004 Krasnodar, Russia; moiseew_a@rambler.ru; 5 Department of Technology of Fats, Cosmetics, Commodity Science, Processes and Devices, Kuban State Technological University, 2 Moscow St., 350072 Krasnodar, Russia; butina_elena@mail.ru

**Keywords:** silver nanoparticles, silk fibers, cyclic freezing, catgut, sorption

## Abstract

This article presents the results of the 10-fold cyclic freezing (−37.0 °C) and thawing (0.0 °C) effect on the number and size range of silver nanoparticles (AgNPs). AgNPs were obtained by the cavitation-diffusion photochemical reduction method and their sorption on the fiber surface of various suture materials, perlon, silk, and catgut, was studied. The distribution of nanoparticles of different diameters before and after the application of the cyclic freezing/thawing processes for each type of fibers studied was determined using electron microscopy. In general, the present study demonstrates the effectiveness of using the technique of 10-fold cyclic freezing. It is applicable to increase the absolute amount of AgNPs on the surface of the suture material with a simultaneous decrease in the size dispersion. It was also found that the application of the developed technique leads to the overwhelming predominance of nanoparticles with 1 to 15 nm diameter on all the investigated fibers. In addition, it was shown that after the application of the freeze/thaw method, the antibacterial activity of silk and catgut suture materials with AgNPs was significantly higher than before their treatment by cyclic freezing.

## 1. Introduction

Methods for preventing the development of the infection directly in the area of surgical intervention are being continuously improved. Although the method of vacuum therapy of wounds plays a special role, including the use of combined antibacterial drugs (primarily in the treatment of postoperative wounds with a high risk of developing a surgical infection [1,2,3,4]), one of the most significant areas of prevention of wound suppuration is the use of formulations based on silver, for example, in the form of nanoparticles [5,6,7]. It should be taken into account that the properties of the obtained nanoparticles will largely change depending on the conditions of their synthesis, physicochemical properties of reducing agents and stabilizers [8,9,10,11]. Therefore, the synthesized silver nanoparticles (AgNPs) can differ significantly in size, sedimentation, and aggregate stability, and, consequently, in antimicrobial activity [12,13,14,15,16].

At the present time, an important direction in the use of AgNPs is their introduction directly into the structure of the suture material (SM). That is due to the development of new high-tech methods of “in situ” processing of artificial and natural polymers. In a number of cases, sutures treated in this way have shown strong antibacterial activity against pathogenic gram-negative and gram-positive bacteria (for example, *Escherichia coli* with an extended spectrum of beta-lactamases, *Staphylococcus aureus*, *Streptococcus Pneumoniae* and others [17,18]), mold, and yeast, and high biocompatibility with fibroblasts, which accelerates the wound healing process, has been revealed. These results indicate that AgNPs are an effective agent against wound infectious complications caused by multidrug-resistant microorganisms [17,18,19,20].

In turn, in a number of studies, the process of sorption of AgNPs on the surface of SMs of various origins was studied [21,22], which demonstrated significant differences in the sorption activity of nanoparticles depending on the structure of individual fibers. It is well known that upregulated antimicrobial and cytotoxic properties of AgNPs are almost always observed for smaller nanoparticles with diameters of about 10 nm and less (compared to the same concentration of AgNPs with a larger average size). Because AgNPs have a bigger summary surface area, silver ions (Ag^+^) can release faster from them and therefore their concentration is usually higher in a wound. Moreover, smaller AgNPs can provide reactivity, including endocytosis and exocytosis, into cellular membranes much easier and quicker. In most cases, similar immobilized silver nanoparticles lead to increased oxidative stress only at the place of their application. This additionally arises the antibacterial effect of them significantly, foremost by binding of sulfhydryl groups (–SH) of bacteria’s enzyme systems [23,24,25]. It is important to point out that AgNPs cytotoxic activity calculated in a human’s body weight is not exiguous enough to cause any health risk [26]. In addition, the biological activity of silver nanoparticles strongly depends on their shapes, for instance, the spherical AgNPs (average size about 10–15 nm) showed far better antibacterial properties than the nanorods, triangular and bigger spherical shaped nanoparticles [23,27]. Nevertheless, in the antibacterial activity test the anisotropic-shaped nanoprisms containing mainly high-atom-density facets (named lattice planes {111} with a range size from 25 to 400 nm) can show much stronger microbicidal properties than spherical nanoparticles [28,29], due to the maximum reactivity of their sharp edges [27,30]. 

A promising direction for further research in this area is the creation of methods aimed at increasing the number of AgNPs of small diameter (from 1 to 10 nm [17,31]) on the surface of the SM, after additional treatment of fibers with sorbed nanoparticles by physical factors, for example, cyclic exposure to temperatures below 0.0 °C. The method of low temperatures with calcium ions (as a cross-linking agent) was previously used to obtain alginate wound dressings containing silver nanoparticles and demonstrated high antibacterial activity [32]. According to recent studies, deep freezing can promote the additional formation of silver nanoparticles in the presence of organic material in the form of specific nanostructures (linear aggregates, nanocomposites, solid structures) [33,34]. Based on this, it can be assumed that cyclic freezing will differently, but at the same time significantly, affect the processes of modification of sorbed nanoparticles and their dynamic equilibrium on the surface of both artificial and natural SM. It may promote the development of more potent AgNPs-based antibacterial agents due to their numerosity “in situ”, broad-spectrum and robust antimicrobial properties, especially against microorganisms with combating multidrug resistance [35,36]. All previously noted can provide far better prevention from infectious diseases and significantly decrease the number of complications, such as reinfection and suppuration after wound suturing.

In connection with the above-mentioned, the purpose of this research was to study the features of the formation of silver-containing nanostructures on the surface of suture material of artificial (perlon) and natural (silk, catgut) origin when performing 10-fold cyclic freezing (in the temperature range from 0.0 to −37.0 °C).

## 2. Materials and Methods

### 2.1. Materials

The preparation of AgNPs was carried out by the method of cavitation-diffusion photochemical reduction (CDPhR method). This method involves a combined complex effect of ultraviolet radiation (wavelength 280 nm) and ultrasound (radiation frequency 1.7 MHz) on the forming nanoparticles for 60 minutes [37,38]. The reduction of silver ions occurred under continuous stirring in the presence of a ligand, polyvinylpyrrolidone (PVP) (average molecular weight 8000 Dalton, from Ltd “AK Sintvita”, Tula, Russia, pharmacopoeia article FSP 42-0345-4367-03). The synthesis of the nanoparticles was carried out by reducing silver ions (Ag^+^) in an aqueous solution in the presence of a stabilizing ligand PVP according to the following four steps. Step 1: the weighed amount of the ligand was prepared and dissolved in bidistilled water until the 1% *w*/*v* solution was obtained. Step 2: 5% *w*/*v* NaOH solution (obtained from chemically pure sodium hydroxide GOST 4328-77, Moscow, Russia) in the volume ratio of 5:1 was added to 1% *w*/*v* AgNO_3_ solution (obtained from chemically pure silver nitrate GOST 1277-75, Moscow, Russia), and the formed Ag_2_O precipitate was washed with bidistilled water. Step 3: the prepared 1% *w*/*v* PVP solution was added to the resulting suspension of Ag_2_O in bidistilled water under vigorous stirring. After the formation of a homogeneous solution, the volume was adjusted with bidistilled water to obtain 0.0059 M silver solution. Step 4: the photochemical reduction of 0.0059 M silver solution was carried out under ultraviolet radiation at the wavelength 280 nm and ultrasound at radiation frequency 1.7 MHz for 60 min. 

To perform the sorption experiment, a freshly prepared solution with nanoparticles was diluted with distilled water to a final concentration of 5 µg/mLAgNPs, thermostated at 60 °C and then gelatin (obtained from gelatin for the medical industry GOST 23058-89, Moscow, Russia) was added to a final concentration of 0.9% *w*/*v* to obtain a gel composition [21].

To assess the dynamics of sorption of AgNPs, materials of metric size 2-0 (or #2/0) were used, including two materials of natural origin: silk, which is a protein fiber consisting of fibroin and sericin [39], and catgut, consisting of collagen filaments [40]. For comparison, we used an SM of artificial origin “Perlon” (nylon-6), which is a polymerization product of ε-caprolactam [41]. Silk was a sterile non-absorbable polyfilament braided thread of natural origin produced by Volot TM (Moscow, Russia). Catgut was presented in the form of a sterile absorbable surgical monofilament suture of natural origin manufactured by Lintex Ltd. (St. Petersburg, Russia). Perlon was used in the form of a non-absorbable multifilament braided surgical suture of artificial origin made by Kerbl East Sp. z o.o. (Wola Rasztowska, Poland).

Sections of the selected SMs 10 mm long were immersed in a gel composition containing silver nanoparticles, then removed and subjected to 10-fold cyclic freezing, consisting of a sequential alternation of freezing to −37.0 °C for 24 h and then raising the temperature to 0 °C for the same period. The cyclic freezing was carried out with the low-temperature freezer “Haier DW-86L288” with an electronic display (Haier Medical and Laboratory products Co., Ltd., Shandong, China).

### 2.2. Characterization Methods

The AgNP number in the colloid was obtained by counting nanoparticles through electron microscopy on a JEOL-7500F scanning electron microscope (SEM) (JEOL, Tokyo, Japan) with a magnification of 250,000x. AgNPs aggregation stability in the solution for SMs incubation was assessed using two electrolyte solutions: 1.0% *w*/*v* NaCl and 5.0% *w*/*v* Na_3_PO_4_ (obtained from chemically pure sodium chloride, GOST 4328-77 and sodium phosphate tribasic dodecahydrate, GOST 201-76, Moscow, Russia, respectively). The optical density of the obtained solution with silver nanoparticles was measured using a KFK-3 photometer (Zagorsk optical and mechanical plant, Sergiev Posad, Russia).

The results of the sorption of silver nanoparticles on the surface of the SMs under investigation were evaluated by SEM with a field emission cathode at an accelerating voltage of 10 kV, in the reflected electron detection mode (COMPO) with a magnification of 30,000× (“Center for collective use of diagnostics of structures and properties of nanomaterials” Kuban State University, Krasnodar, Russia). The assessment of the size distribution of nanoparticles was carried out according to the generally accepted way by measuring the diameter of all AgNPs in the SEM image area at the magnification of 30,000× [42,43,44].

The ability of the fibers with sorbed AgNPs, obtained by the CDPhR method, to inhibit microbial growth was evaluated through a zone of inhibition test (Kirby-Bauer test). This is a common qualitative method widely used in clinical trials as a quick and easy way to measure antimicrobial ability of medical antibacterial-impregnated textiles and compare their levels of inhibitory activity. Using a sterile swab, a suspension of the pure culture of *Escherichia coli* (*E. coli 25922*) was spread evenly over the face of a sterile agar plate (containing Mueller-Hinton agar). The antimicrobial fibers with AgNPs and control fibers without AgNPs were placed in the agar plate. The agar plate was incubated for 24 h at the temperature of 37 °C, then the size of the clear zone (or the zone of inhibition, which is related to the level of antimicrobial activity of the fiber) around each test sample was measured.

### 2.3. Statistics

Statistical processing of the obtained data was carried out on specialized software using the Kruskal-Wallis nonparametric analysis of variance (H-test–Kruskal-Wallis ANOVA). Differences were considered significant at *p* < 0.05. Data in the graphs are presented as median (Me), percentile-25 (P_25_), and percentile-75 (P_75_).

## 3. Results

**Figure 1:**
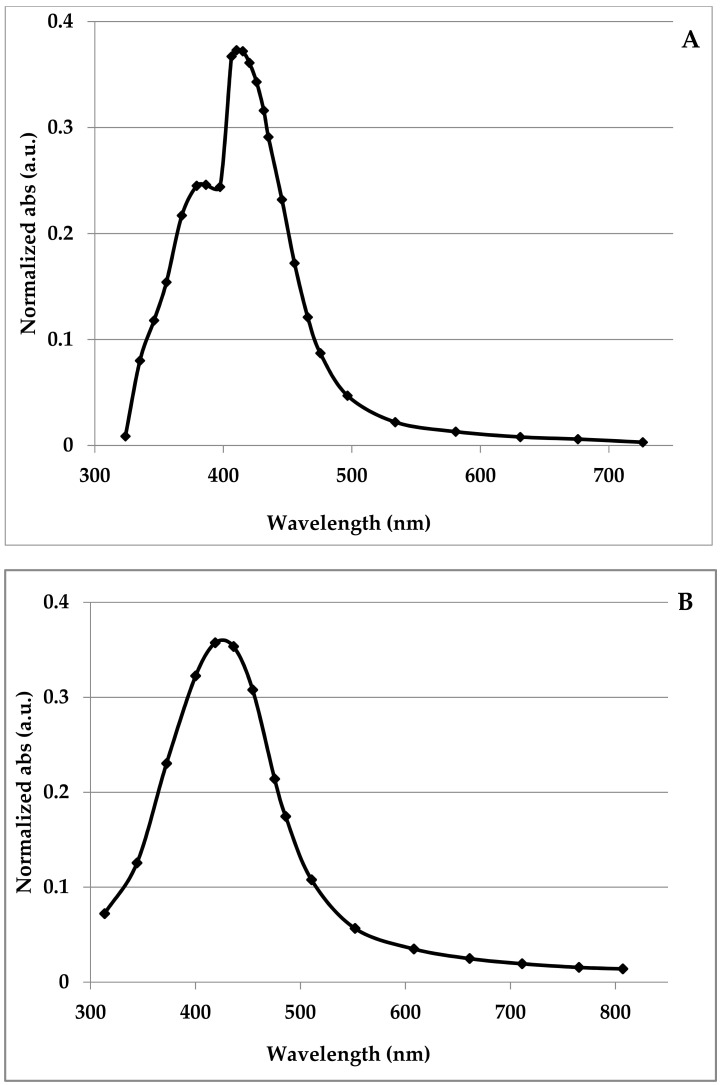
Ultraviolet-visible absorption spectra of the AgNPs solution. (**A**) immediately after nanoparticle synthesis; (**B**) after a month.

**Figure 2:**
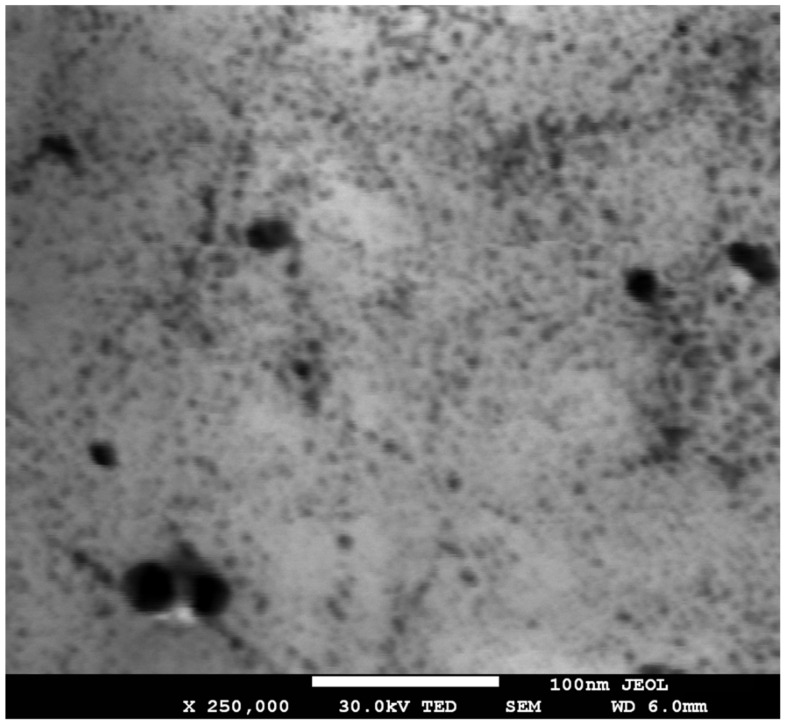
SEM photograph of AgNPs solution (immediately after nanoparticle synthesis).

The optical properties of the silver nanoparticles solution showed that the absorption peak of the optical density, measured on a photometer, was in the visible region at around 410 nm (Figure 1A) and remained practically unchanged for a month since the initial synthesis (Figure 1B). This result also indicates the significant resistance of obtained AgNPs to their spontaneous and irreversible formation of irregular particle assemblages or agglomerates during storage. This absorption peak and the greenish-yellow color of the AgNPs solution (Figure S1) indicated that the nanoparticles were mainly spherical with some range of size distribution evaluated mainly via electron microscopy (68% of AgNPs had size less than 6 nm, 23% from 6 to 10 nm, 9% more than 10 nm, Figure 2). This data generally correlates with Gustav Mie’s theory and its basic modifications and it is relevant to the shift of plasmon resonance maximum in the solutions with small spherical particles when no coherent phase relations among the scattered light from different spheres due to the distance of large enough between them in the stable colloid [45,46]. Moreover, it was found that the obtained silver nanoparticles were totally stable in NaCl, with concentrations of 1.0 and 5.0% *w*/*v* and Na_3_PO_4_, concentrations of 1.0 and 5.0% *w*/*v*, solutions. These results indicate that the aggregate stability of silver nanoparticle solution, obtained using the CDPhR method, is good for medical applications. In addition, the small shoulder in the range from 379 to 386 nm (Figure 1A) could indicate the presence of both some number of AgNPs with a diameter less than 1 nm or a small amount of the non-spherical clusters of nanoparticles [47,48].

**Figure 3:**
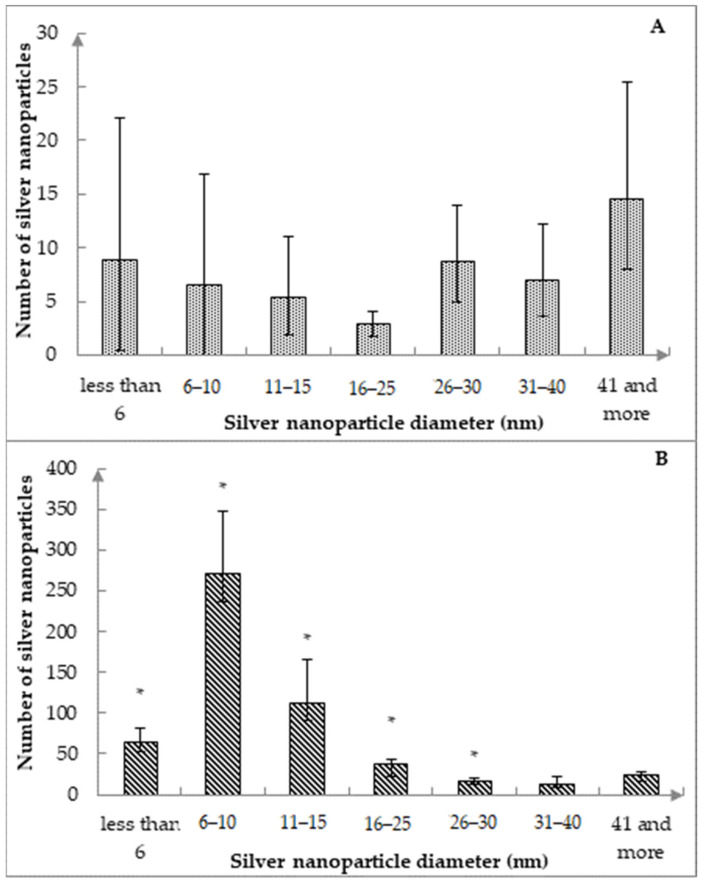
Distribution of silver nanoparticles on the surface of catgut suture material without exposure to temperatures below 0.0 °C (**A**) and after 10-fold cyclic freezing (**B**). Note: *—*p* < 0.05, in comparison with the same size range before cyclic freezing; data are presented as Me, P_25_, P_75_.

**Figure 4:**
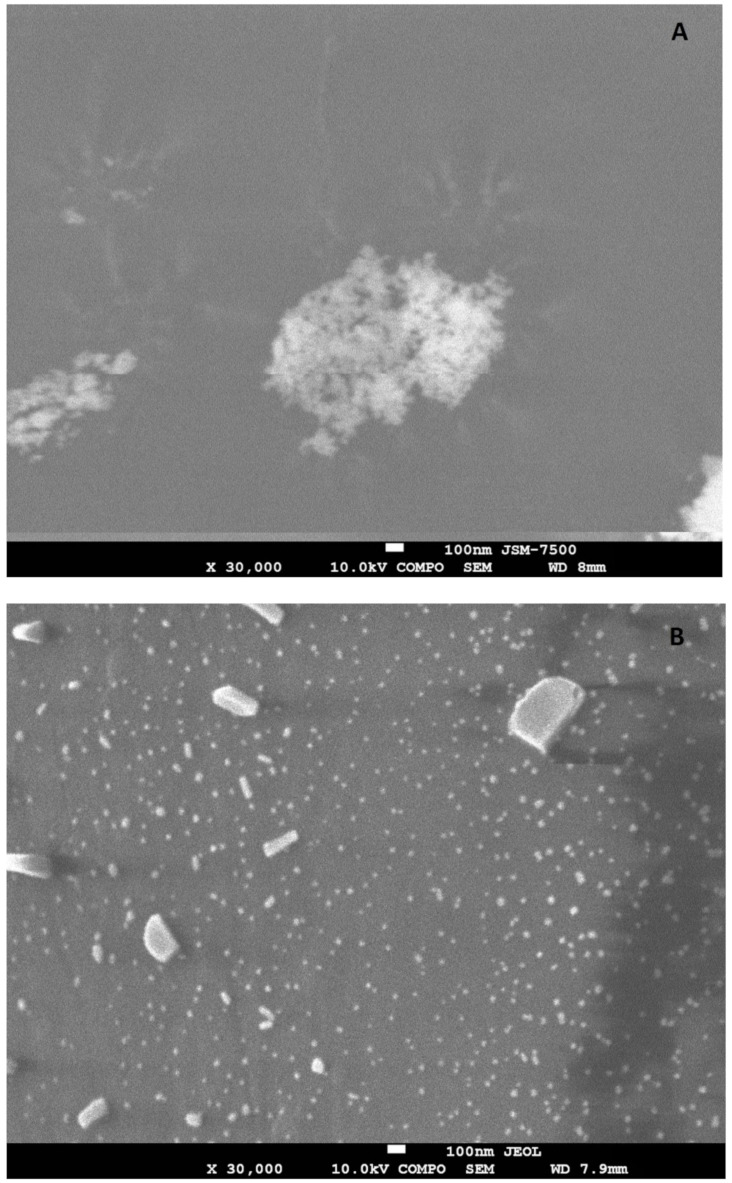
SEM photographs of silver nanoparticles on the surface of the catgut suture material: (**A**) before cyclic freezing/thawing; (**B**) after 10-fold cyclic freezing/thawing.

In absence of exposure to subzero temperatures, the following size intervals prevail (*p* < 0.01773) on catgut fibers (CFs): less than 6 nm (16.4%), from 26 to 30 nm (16.3%), and especially 41 nm and more (26.8%). After the cyclic freezing/thawing processes, it was found that silver nanoparticles were distributed more uniformly on the surface of the CFs (Figure 3B), as also shown in the SEM photographs (Figure 4A,B). There is a clear shift toward more AgNPs in the diameter range of less than 16 nm. At the same time, the largest number of AgNPs was detected in the 6–10 nm size range, which was 4.3 times, 2.4 times, 7.1 times, 16.0 times, 19.9 times, 11.4 times higher compared to the ranges less than 6 nm, 11–15 nm, 16–25 nm, 26–30 nm, 31–40 nm, 41 nm and more, respectively, (*p* < 0.00002).

Despite an increase in the total number of nanoparticles of all sizes after cyclic freezing by more than 9.9 times (*p* < 0.05), no significant differences were found in the ratio of silver nanoparticles with sizes from 31 to 40 nm (*p* > 0.065), 41 nm and more (*p* > 0.336). This result indicates an increase in the number of AgNPs after the cyclic freezing process on catgut exclusively for nanoparticles of small and medium diameters (i.e., no more than 30 nm, Figure 3B).

Compared to the number of AgNPs obtained on the same CF without additional heat treatment, there was a significant difference in the size distribution of nanoparticles after cyclic freezing between the number of AgNPs with similar diameters in the range of less than 31 nm (Figure 3A,B). At the same time, after 10-fold freezing/thawing, the absolute number of nanoparticles for each diameter from the specified size range increased 1.9–41.4 times (the least significant for AgNPs with a diameter from 26 to 30 nm, the most significant for AgNPs with a diameter from 6 to 10 nm).

**Figure 5:**
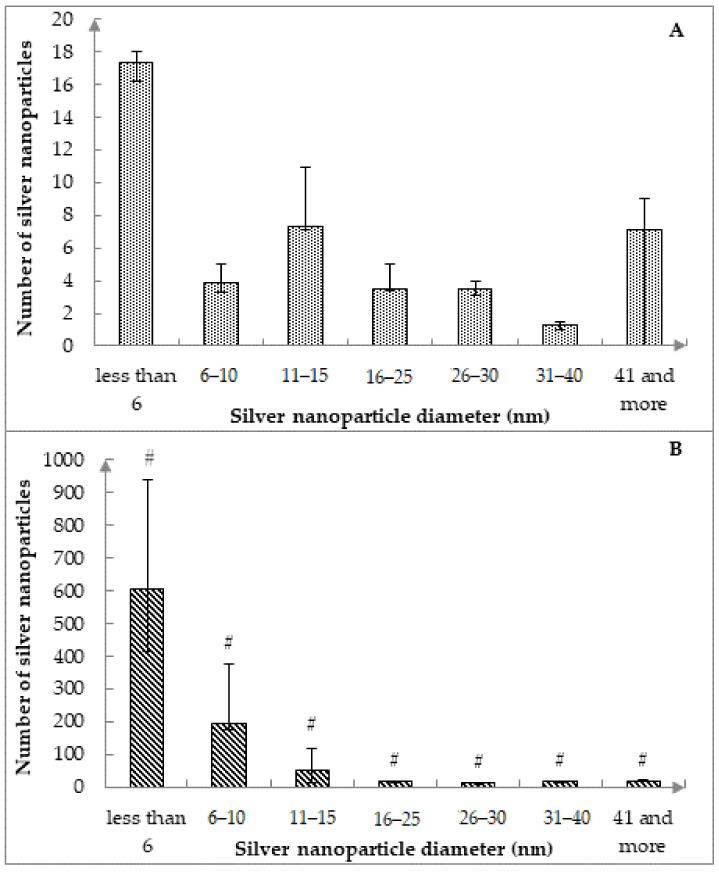
Distribution of silver nanoparticles on the surface of perlon suture material without exposure to temperatures below 0.0 °C (**A**) and after 10-fold cyclic freezing (**B**). Note: #—*p* < 0.05, compared to the same size range before cyclic freezing; data are presented as Me, P_25_, P_75_.

**Figure 6:**
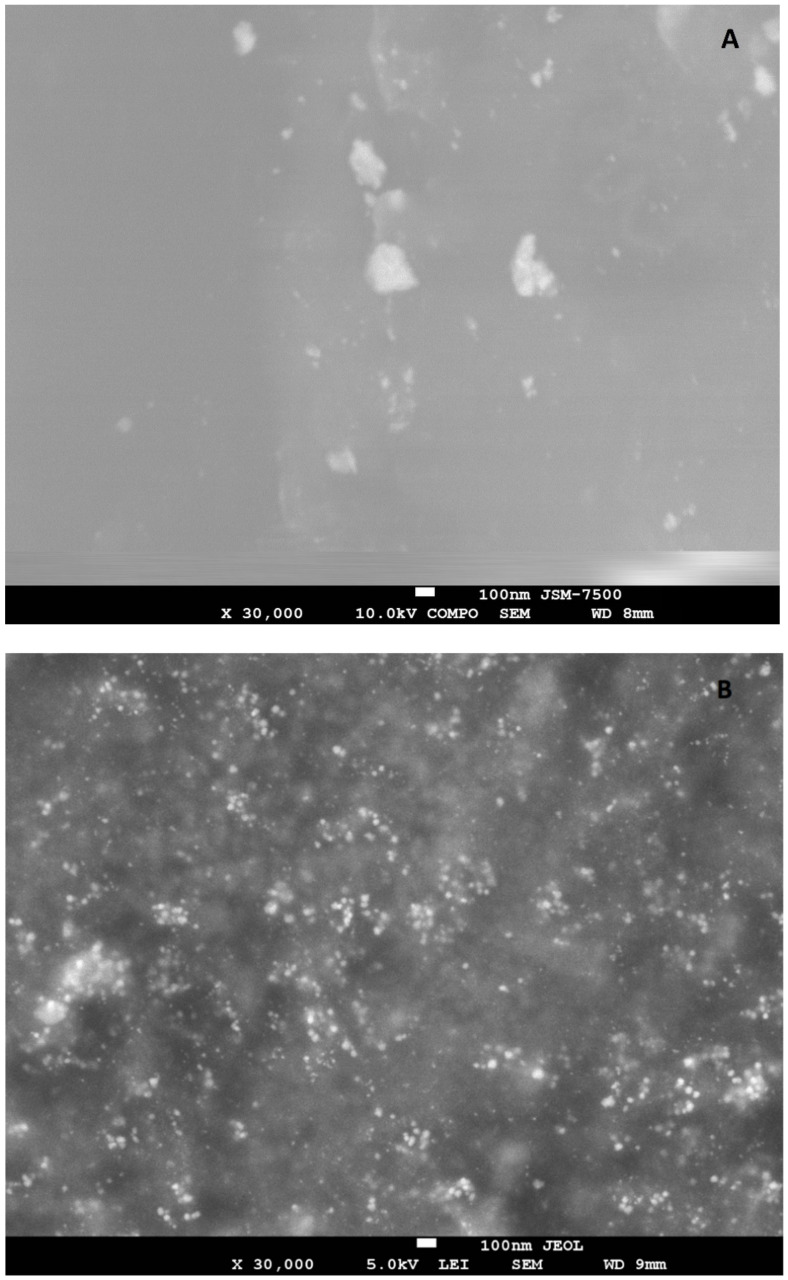
SEM photographs of silver nanoparticles on the surface of the perlon suture material: (**A**) before cyclic freezing/thawing; (**B**) after 10-fold cyclic freezing/thawing.

On the surface of the perlon suture fibers (PFs), the prevalence of the number of silver nanoparticles of the following size ranges (*p* < 0.00002, Figure 5A) was revealed: less than 6 nm (39.4%), 11–15 nm (16.7%), 41 nm and more (16.1%). Although the total number of nanoparticles of all sizes on the perlon surface before cyclic freezing was 18.6% lower than on CFs, the number of AgNPs with 1 to 5 nm diameter on PFs was 2.0 times higher (*p* < 0.05, Figure 3A, Figure 5A and Figure 6A).

After cyclic freezing, an increase in the number of silver nanoparticles of all size ranges was also revealed on the surface of the perlon SM fibers ( Figure 5B and Figure 6B). In addition, after cyclic freezing/thawing on PFs, a greater number of AgNPs of all sizes (by 70.9%) was revealed in comparison with catgut, primarily due to a separate fraction of nanoparticles with a diameter of less than 6 nm, which was 9.5 times higher on PFs, compared with catgut (*p* < 0.0015).

Compared to the number of AgNPs detected before the cyclic freezing/thawing, after the temperature treatment of perlon, a pronounced increase in the number of nanoparticles of all the following diameters was noted: for AgNPs with diameter less than 6 nm, by 34.8 times (*p* < 0.005), from 6 to 10 nm by 50.7 times (*p* < 0.004), from 11 to 15 nm by 7.3 times (*p* < 0.006), from 16 to 25 nm by 4.8 times (*p* < 0.01), from 26 to 30 nm by 3.8 times (*p* < 0.004), from 31 to 40 nm by 14.7 times (*p* < 0.005), 41 nm and more by 2.6 times (*p* < 0.01, Figure 5 A,B). Moreover, after cyclic freezing, a significant prevalence of the number of nanoparticles with a diameter from 1 to 5 nm over other size ranges in 3.1–45.3 times was noted (the least significant over AgNPs with a diameter from 6 to 10 nm, the most significant over AgNPs with a diameter from 26 to 30 nm, *p* < 0.00028, Figure 5B).

**Figure 7:**
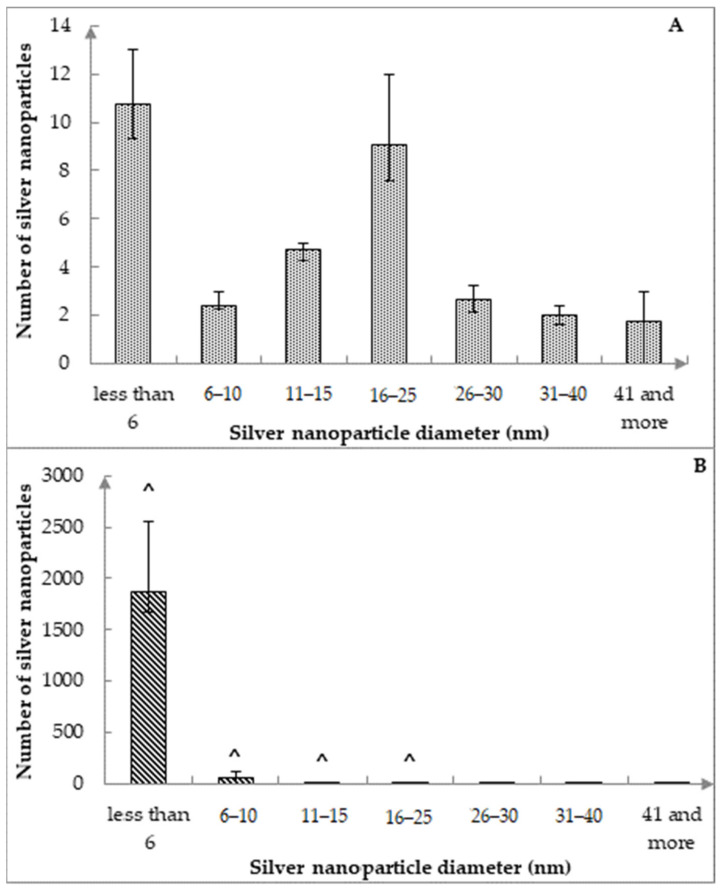
Distribution of silver nanoparticles on the surface of silk without exposure to temperatures below 0.0 °C (**A**) and after 10-fold cyclic freezing (**B**). Note: ^—*p* < 0.05, compared to the corresponding size range before cyclic freezing; data are presented as Me, P_25_, P_75_.

On the surface of the perlon SM fibers, the predominance of AgNPs of the following size ranges (*p* < 0.00003, Figure 7A) was revealed: less than 6 nm (32.3%), 16–25 nm (27.2%), and 11–15 nm (14.1%). At the same time, the total number of nanoparticles on the surface of silk fibers (SFs) was 38.1% lower than the number on the surface of CFs (Figure 3A and Figure 7A), primarily due to the lower content of AgNPs on SFs in comparison with catgut in the range above 25 nm, including 41 nm and more in diameter (87.8% lower), 31 to 40 nm in diameter (71.4% lower) and 26 to 30 nm in diameter (69.5% lower). 

On the surface of silk threads after cyclic freezing, the most significant increase in the number of AgNPs with a diameter less than 6 nm was found in comparison with similar indicators before exposure to a temperature of −37.0 °C (by a factor of 174.0, *p* < 0.004, Figure 7A and Figure S2). Moreover, significantly higher values of AgNPs were found after freezing/thawing in comparison with the indicators before the temperature treatment of silk for particles with a diameter from 6 to 10 nm (23.2 times, *p* < 0.05). At the same time, after cyclic exposure to a temperature of −37.0 °C on SFs with AgNPs, a decrease in the number of nanoparticles with a diameter from 11 to 15 nm (by 81.4%, *p* < 0.03) and from 16 to 25 nm (by 96.9%, *p* < 0.004, Figure 7A,B) was observed. It should be noted that the number of nanoparticles with a size of 26 nm and more on SFs did not change significantly after cyclic exposure to subzero temperatures (*p* > 0.05, Figure 7B). In addition, after cyclic freezing/thawing, AgNPs with a diameter less than 6 nm on SFs accounted for 96.7% of the total number of nanoparticles and significantly prevailed over all ranges (34.0–6693.4 times, *p* < 0.00092, Figure 7B and Figure S2).

At the same time, among all the studied fibers, the largest number of AgNPs with a diameter less than 6 nm after 10-fold cyclic freezing was formed on silk threads and exceeded the similar indicators on catgut and perlon by 29.4 times and 3.1 times, respectively, (*p* < 0.002). At the same time, most AgNPs with a diameter from 6 to 10 nm after cyclic exposure to subzero temperatures (−37.0 °C) were formed on CFs and exceeded the similar parameters on silk and perlon by 4.9 times and 1.4 times, respectively, (*p* < 0.006). Furthermore, the content of AgNPs on SFs was significantly lower compared to similar indicators on catgut and perlon among larger nanoparticles (11 nm and more); the smallest *p* was 0.0011 when comparing AgNPs with a diameter of 16 to 25 nm, the highest *p* was 0.0071 when comparing AgNPs with a diameter of 41 nm and more (Figure 3B, Figure 5B and Figure 7B). It should be pointed out that the number of nanoparticles on the three investigated fibers before the cyclic freezing procedure also significantly differed when comparing the sizes of AgNPs in the ranges from 16 nm and more (Figure 3A, Figure 5A and Figure 7A). The number of nanoparticles with a diameter of 16 to 25 nm on silk was 2.6 times and 3.1 times higher than on perlon and catgut, respectively, (*p* = 0.0264); the diameter from 26 to 30 nm on catgut was 3.3 times and 2.5 times higher than on silk and perlon, respectively, (*p* = 0.0366); diameter from 31 to 40 nm on catgut was 5.4 times and 3.5 times higher than on perlon and silk, respectively, (*p* = 0.027); diameter of 41 nm and more on catgut was 8.2 times and 2.0 times higher than on silk and perlon, respectively, (*p* = 0.0059).

**Figure 8:**
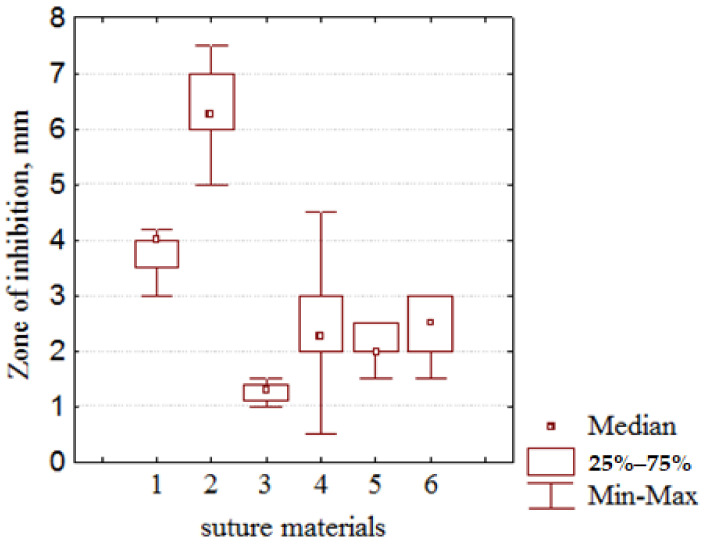
Changes in the antibacterial activity of the different fibers with AgNPs after cyclic freezing/thawing: 1: silk fibers with AgNPs and without cyclic freezing/thawing; 2: silk fibers with AgNPs and with cyclic freezing/thawing; 3: the catgut fibers with AgNPs and without cyclic freezing/thawing; 4: catgut fibers with AgNPs and with cyclic freezing/thawing; 5: perlon fibers with AgNPs and without cyclic freezing/thawing; 6: perlon fibers with AgNPs and with cyclic freezing/thawing.

**Figure 9:**
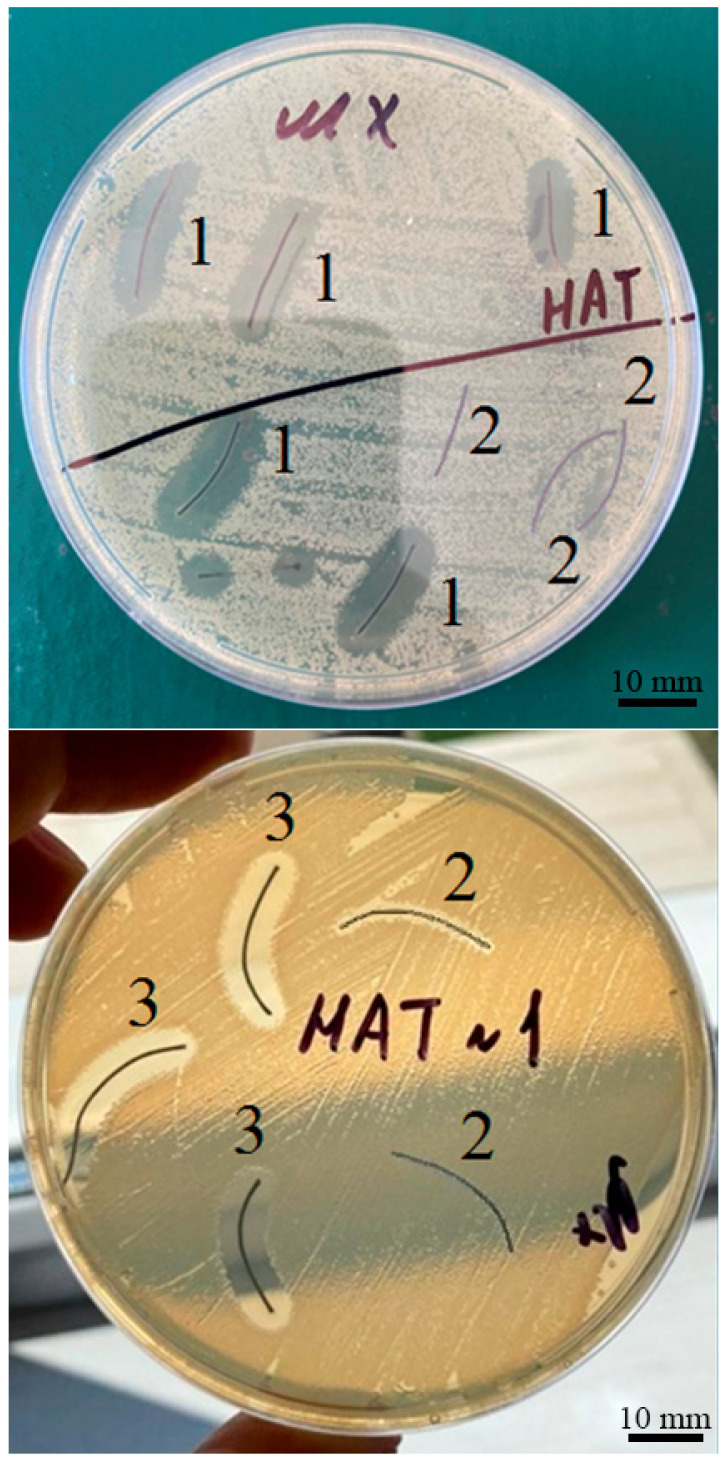
Inhibitory effect of silk fibers with AgNPs and with cyclic freezing/thawing (1) without AgNPs (2) with AgNPs and without cyclic freezing/thawing (3) against *Escherichia coli* bacteria by means of disc diffusion.

In addition, before cyclic freezing, the most antibacterial activity was found for the silk fibers, which exceeded the antibacterial activity of the catgut and perlon fibers by 3.1 and 2.0 times, respectively, (*p* < 0.0001, Figure 8). After using the method of freezing/thawing the zone of the bacterial growth, inhibition by the silk threads was higher by 1.5 times (*p* < 0.0001, Figure 8, Figure 9 and Figure S3) and the antibacterial activity of the catgut threads was higher by 1.7 times (*p* = 0.0184, Figure 8), but the last still was much lower (2.8 times, *p* < 0.0002, Figure 8) in comparison with the antibacterial activity of the SFs after applying the cyclic freezing. Exposure to bacterial growth of PFs samples (after a 10-fold freeze/thaw cycle) increased antibacterial activity by 1.25 times compared to original PFs samples (*p* = 0.112, Figure 8). It should be noted that, if before using the 10-fold freezing/thawing, the antimicrobial activity of the CFs was strongly lower (by 65%, *p* < 0.0003, Figure 8) than the PFs, after cyclic freezing this index was almost the same for both these SMs: perlon (100%) ≈ catgut (90%) with the *p*-value equaled to 0.726. 

## 4. Discussion

Both the multiple increases in the number of nanoparticles with a diameter of 16 nm on the surface of all studied samples (silk > perlon > catgut) and the significant change in the distribution of nanoparticles of bigger sizes after 10-fold thawing may indicate the additional formation of AgNPs on the surface of the materials during cyclic freezing (Figure 3, Figure 5 and Figure 7). In general, such a result can be associated with the interaction of silver ions with functional groups in the fiber composition. For example, the interaction of AgNPs and Ag^+^ in catgut can occur with carboxyl (–COOH), hydroxyl (–OH), and nitrogen-containing heterocyclic amino acid side radicals (tryptophan [49,50,51], histidine [52], proline [53,54]). The sorption of silver-containing particles by perlon fibers, which is a product of caprolactam polymerization, is largely due to the binding of AgNPs and Ag^+^ with carbonyl groups (C=O) in the fiber composition [50]. The advantage of silk threads is the presence of an organic component in their composition, which is necessary to accelerate the recovery processes during freezing. Silk is known to contain sericin, and AgNPs have a high affinity for it. In addition, sericin has cytocompatibility with mammalian cells and is able to retain AgNPs, as well as efficiently release them directly into the wound [55]. Moreover, sericin-conjugated AgNPs retain their high antibacterial activity against *Escherichia coli* (12–15 mm zone of inhibition), *Staphylococcus aureus* (14.6–15.4 mm zone of inhibition), and *Klebsiella pneumoniae* (12.5–18 mm zone of inhibition) in environments with different pH levels (3.0–11.0) and temperatures (4–55 °C), which allows them to be used as an antimicrobial agent [56]. The interaction of silver and sericin can occur due to the hydroxyl group (−OH) of tyrosine, which can manifest itself as a specific signal in FTIR-spectroscopy at 1640cm^−1^ [56], as well as by van der Waals interaction with stabilization of the structure of the [AgNP–protein] complex due to hydrophobic regions leading to an increase in the binding constant with increased temperature [51]. The formation of the [AgNP–protein] complex also provides additional energy transfer between amino acids (tyrosine, tryptophan, phenylalanine) and silver nanoparticles, which can play a role in the regeneration of silver ions during cyclic freezing/thawing of fibers with previously adsorbed nanoparticles.

The obtained results indicate both the different sorption activity of AgNPs under the same conditions, depending on the properties of the SM and its structure at temperatures above 0.0 °C, and the effect of the structure and chemical composition of fibers on the number of nanoparticles after 10-fold cyclic freezing/thawing. The latter may be due to different rates of hydrothermal reduction of Ag^+^ ions and different intensities of intermolecular interaction of AgNPs (including active chemical groups of both a ligand, for example, polyvinylpyrrolidone, and a directly sorbing polymer), upon repeated exposure to a temperature of −37.0 °C on fibers with sorbed nanoparticles. Thus, the selective formation of nanoparticles up to 10 nm in diameter on various polymers is ensured by exposing them to low temperatures in the “AgNPs-Ag^+^-fiber” system. At the same time, this occurred most significantly on silk fibers (Figure 7B), and was less pronounced on perlon and catgut, but with the preservation of the general trend (the number of AgNPs before freezing < the number of AgNPs after freezing). Indeed, as was established in another study, the formed hierarchical structures depend not only on the nature of the substrate but also on the activity of the applied components [57,58]; therefore, the use of cyclic freezing can significantly stimulate the synthesis of new nanoparticles.

In this regard, the formation of a larger number of nanoparticles up to 10 nm after freezing can be explained by the specific interaction of AgNPs obtained by the CDPhR method with the surface of the studied fibers. This allows us to consider them as a potential reservoir with a higher microbicidal activity when performing medical procedures on infectious-contaminated biological objects [59].

Simultaneously with an increase in nanoparticles less than 10 nm in diameter, only on silk fibers, there is a decrease in the number of AgNPs with a diameter of 11 to 30 nm, which may be associated with desorption processes due to the weakening of the interaction of larger nanoparticles with hydroxyl and carbonyl groups in the composition of sericin. Given the certain fragility of sericin, the additional release of AgNPs due to desorption after cyclic freezing/thawing can serve as an additional source of Ag^+^ and reducing groups (–OH groups of tyrosine in the composition of sericin) required for the in situ formation of new nanoparticles of smaller size (less than 11 nm). 

It is important to note that when using the developed technique of 10-fold cyclic freezing/thawing for individual specific fibers, for example, sericin-containing ones, it is possible to achieve the predominant formation of AgNPs with a diameter of less than 6 nm (up to 96.7%), which undoubtedly increases the possibility of regulating the size of nanoparticles on medical devices, expanding the possibilities of their application in practice [58,60,61], including increasing the antibacterial activity [62] due to their better stability and high biocompatibility with sericin. 

Moreover, the significant rise of the antibacterial activity of the silk and catgut threads by 3.1 and 2.0 times, respectively, (*p* < 0.0001, Figure 8 and Figure 9) after cyclic freezing unambiguously indicates the efficacy of the developed method for application in medical purposes to fight against bacterial infection in the surgical field. Despite the increased number of the AgNPs with a diameter of less than 16 nm, the insignificant change of the bacterial growth inhibition zone for PFs after the method of freezing/thawing obviously shows that, in addition to the big number of nanoparticles, the possibility of their diffusion from SMs into the wound environment is much more influential. This can be explained thanks to the results of a Kirby–Bauer test: the larger sizes of the zones of inhibition microbial growth clearly point out much more Ag^+^ ions diffusion in an agar from the silk and catgut fibers with absorbed AgNPs with respect to the perlon threads. Further, AgNPs on perlon threads can apparently bind stronger by the active groups (including carbonyl groups, or =C=O [50]) present in the perlon fiber composition. In addition, in this case, the increasing of the antibacterial activity of catgut to the level of perlon after freezing/thawing indicates that the developed method of cyclic freezing can effectively improve it for certain surgical materials with some original disadvantages concerning their antimicrobial effects. In this regard, using the developed freezing/thawing of the silk and catgut with AgNPs for surgery purposes is far more promising and reasonable in comparison with the perlon processed by the silver nanoparticles obtained by the CDPhR method.

In general, on the basis of the study carried out, an assessment of the effect of 10-fold cyclic freezing on the formation of silver nanoparticles on the surface of artificial (perlon) and natural (silk and catgut) suture fibers was made, establishing the following basic results:

- The increase in the number of nanoparticles on all suture materials investigated (silk, catgut, perlon) was observed when using cyclic freezing/thawing;
- After cyclic freezing/thawing, AgNPs are distributed more homogeneously in size, while their number significantly predominates in the diameter range less than 16 nm: silk (99.5%) > perlon (92.7%) > catgut (82.9%);
- The largest number of AgNPs with a diameter less than 6 nm after cyclic freezing/thawing was observed on silk fibers (96.7% of the total number of nanoparticles) and significantly prevailed over the same indicator on catgut and perlon;
- The highest absolute and relative number of AgNPs with a diameter of more than 15 nm after cyclic freezing/thawing was observed on catgut;
- The antibacterial activity of the silk fibers with AgNPs before and after cyclic freezing was higher than the perlon and catgut threads with nanoparticles (the zone of the bacterial growth inhibition before cyclic freezing was: silk > perlon > catgut as 100% > 50% > 32%, and after cyclic freezing it was: silk > perlon ≈ catgut as 100% > 40% ≈ 36%);
- Differently from perlon threads with AgNPs, the antibacterial activities of the silk and catgut SMs with nanoparticles were significantly higher (1.5 and 1.7 times, respectively), after cyclic freezing.

## 5. Conclusions

In the present study, we found that 10-fold cyclic freezing (down to −37.0 °C) significantly enhances the formation of AgNPs obtained by the CDPhR method on the surface of the studied SM of artificial and natural origin. The largest increase in the number of nanoparticles was observed on silk and catgut threads. The application of the developed technique leads to the overwhelming predominance of nanoparticles with a diameter of 1 to 15 nm, which can be used in medical practice for additional processing of fibers with AgNPs and for obtaining an SM containing mainly small-diameter nanoparticles. A feature of silk (sericin-containing) fibers is, first of all, the dominance of AgNPs with a diameter of less than 6 nm on them after 10-fold cyclic freezing, which makes it possible to position silk in comparison with perlon and catgut as the most promising material for producing fibers with adsorbed nanoparticles. Moreover, the antibacterial activity of the silk fibers with AgNPs before and after cyclic freezing was higher than perlon and catgut threads with nanoparticles. In addition, the antibacterial activities of the treated catgut SMs with nanoparticles were also significantly higher after cyclic freezing.

## Data Availability

Not applicable.

## Supplementary Materials

The following supporting information can be downloaded at: https://www.mdpi.com/article/10.3390/nano12071164/s1, Figure S1: Color of the AgNPs solutions (1—immediately after nanoparticle synthesis; 2—after two weeks; 3—after a month); Figure S2: SEM photographs of silver nanoparticles on the surface of the silk suture material: A—before cyclic freezing/thawing; B—after 10-fold cyclic freezing/thawing; Figure S3: Inhibitory effect of catgut fibers with AgNPs and with cyclic freezing/thawing (1) without AgNPs (2) with AgNPs and without cyclic freezing/thawing (3) against Escherichia coli bacteria by means of disc diffusion.
