# Supplementary material for: Changes in Number and Antibacterial Activity of Silver Nanoparticles on the Surface of Suture Materials during Cyclic Freezing
Source: Nanomaterials (Basel). 2022 Mar 31;12(7):1164. doi: 10.3390/nano12071164 (PMC9000594; doi:10.3390/nano12071164)
Supplement: Supplementary file 1 [file nanomaterials-12-01164-s001.zip › nanomaterials-1544924-supplementary.pdf]

Supplementary Materials

Changes in Number and Antibacterial Activity of Silver Nanoparticles on the Surface of Suture Materials during Cyclic Freezing

Alexander Basov [1,2], Stepan Dzhimak [2,3], Mikhail Sokolov [2], Vadim Malyshko [1,3], Arkadii Moiseev [4], Elena Butina [5], Anna Elkina [2,3,*] and Mikhail Baryshev [2,3,5]

1 Department of Fundamental and Clinical Biochemistry, Kuban State Medical University, 4 Mitrofan Sedina st., 350063 Krasnodar, Russia; son_sunytch79@mail.ru (A.B.); Intro-2@rambler.ru (V.M.)
2 Department of Radiophysics and Nanothechnology, Kuban State University, 149 Stavropolskaya st., 350040 Krasnodar, Russia; jimack@mail.ru (S.D.); sokolovme@mail.ru (M.S.); baryshev_mg@mail.ru (M.B.)
3 Laboratory of Problems of Stable Isotope Spreading in Living Systems, Federal Research Center the Southern Scientific Center of the Russian Academy of Sciences, 41 Chekhov Ave., 344006 Rostov-on-Don, Russia
4 Department of Organization and Support of Scientific Activities, Kuban State Agrarian University, 13 Kalinina st, 350004 Krasnodar, Russia; moiseew_a@rambler.ru
5 Department of Technology of Fats, Cosmetics, Commodity Science, Processes and Devices, Kuban State Technological University, 2 Moscow st., 350072 Krasnodar, Russia; butina_elena@mail.ru
* Correspondence: anna013194@mail.ru; Tel.: +7-918-068-83-81

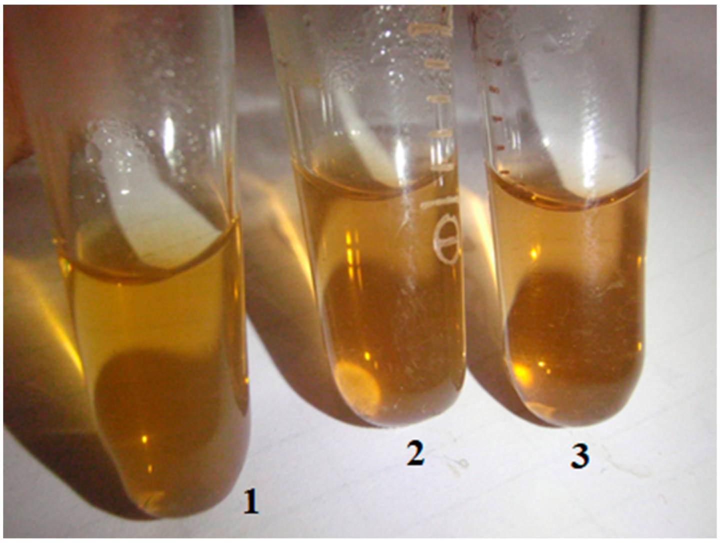

Figure S1. Color of the AgNPs solutions (1 – immediately after nanoparticle synthesis; 2 – after two weeks; 3 – after a month).

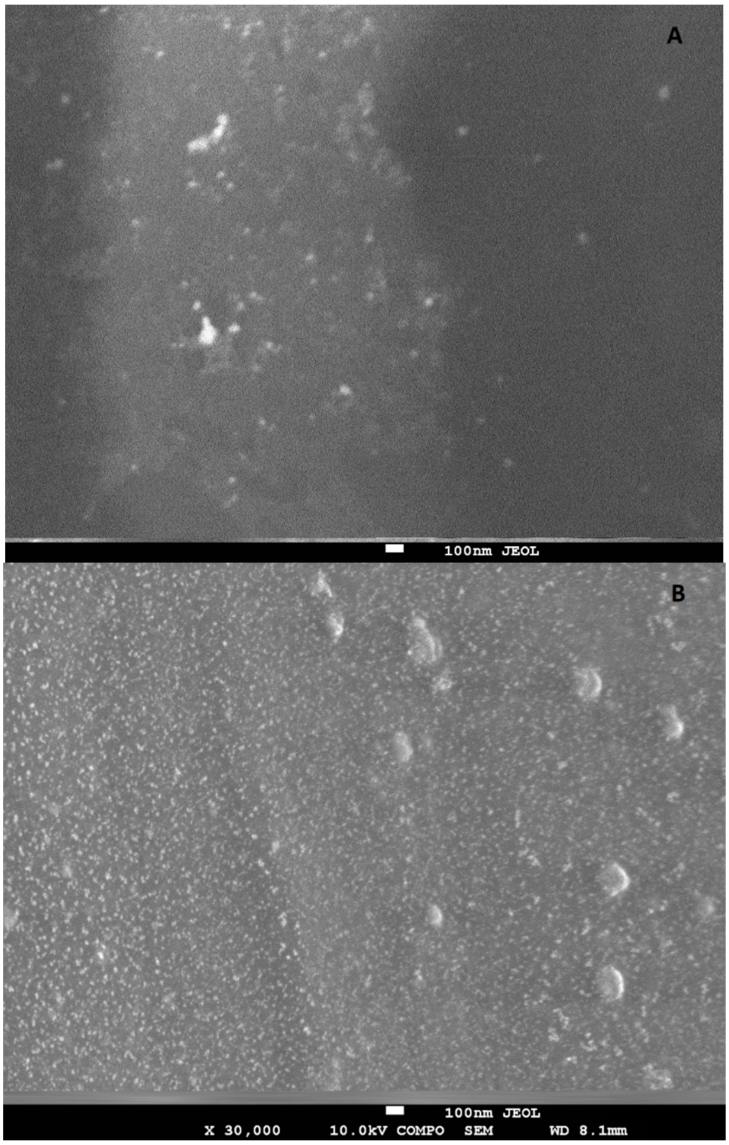

Figure S2. SEM photographs of silver nanoparticles on the surface of the silk suture material: **A** – before cyclic freezing/thawing; **B** – after 10-fold cyclic freezing/thawing

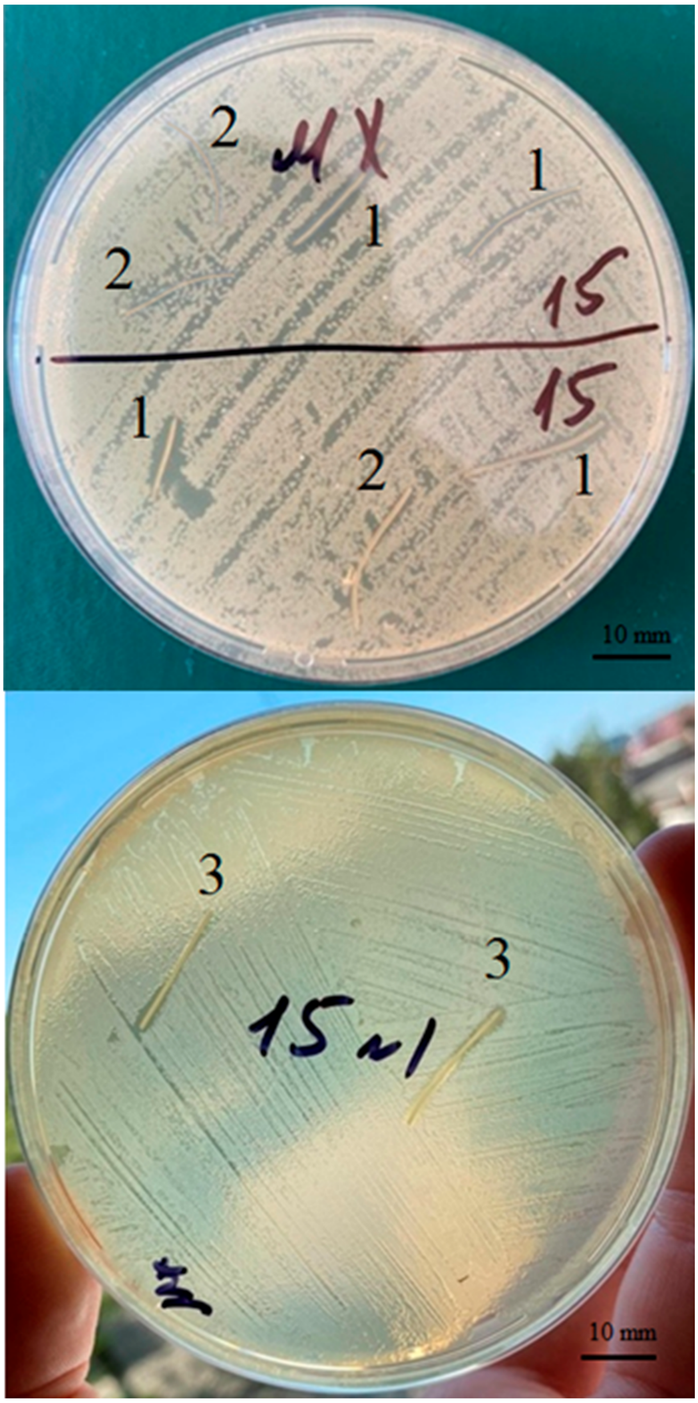

Figure S3. Inhibitory effect of catgut fibers with AgNPs and with cyclic freezing/thawing (1) without AgNPs (2) with AgNPs and without cyclic freezing/thawing (3) against *Escherichia coli* bacteria by means of disc diffusion
